# Beyond 'push–pull': unraveling the ecological pleiotropy of plant volatile organic compounds for sustainable crop pest management

**DOI:** 10.1007/s44297-023-00018-5

**Published:** 2023-12-11

**Authors:** Asim Munawar, Zengrong Zhu, Ricardo A. R. Machado, Wenwu Zhou

**Affiliations:** 1 Ministry of Agricultural and Rural Affairs Key Laboratory of Molecular Biology of Crop Pathogens and Insect Pests, Institute of Insect Sciences, Zhejiang University, Hangzhou, 310058 China; 2 Hainan Institute, Zhejiang University, Sanya, 572000 China; 3 Experimental Biology Research Group, Institute of Biology, University of Neuchatel, 2000 Neuchatel, Switzerland

**Keywords:** Pleiotropy, Plant volatile organic compounds, Crop protection, Plant defense, Sustainable agriculture

## Abstract

Plants produce an array of different volatile organic compounds (VOCs) that have diverse eco-physiological functions and agricultural applications. Hence, the potential of VOCs as a natural and eco-friendly solution to increase crop productivity has garnered substantial attention. In particular, VOC-based pest management measures, such as Push–Pull, have been widely investigated in different cropping systems. However, our understanding of how the release and use of these VOCs impact agro-ecosystems beyond the targeted effects is still very limited. Therefore, studying the functions of plant VOCs from the perspective of pleiotropy may offer insights into optimizing and improving the effectiveness of VOC-based agronomic practices. Here, we discuss the current literature on the pleiotropic functions of plant VOCs, illustrate the underlying mechanism for their production and propose a systematic perspective for the usage of plant VOCs to enhance the sustainable management of crop health.

## Introduction

Plants interact with other organisms in nature using numerous chemicals [1–6]. Volatile organic compounds (VOCs) are among the most well-characterized chemical cues that have a significant role in facilitating the interactions between plants and their surrounding environment [7]. Plant VOCs are a large group of lipophilic hydrocarbon compounds derived from different biosynthetic pathways. They are commonly categorized into terpenoids, benzenoids/phenylpropanoids, fatty acid derivatives and amino acid derivatives according to their origins [8]. These low molecular weight organic compounds are released into the environment rapidly upon synthesis, potentially affecting the various surrounding organisms in natural ecosystems. Hence, the experimental manipulation and release of VOCs in terrestrial ecosystems can exert a substantial impact on the performance of many organisms and interconnected food webs [9]. In agriculture, the “Push–Pull” strategy acts as a successful example of pest management that relies on the ecological function of plant VOCs in the agro-ecosystem [10]. It consists of intercropping crops with other plant species so that these companion plants can release a combination of attractive and repulsive VOCs, influencing the population dynamics of insect species [11, 12]. For example, intercropping cucumber with *Malabar spinach* or *Apium graveolens* companion plants results in a decrease in the population of whiteflies on cucumber plants. D-limonene and geranyl nitrile were identified as the two major VOCs emitted from these companion plants. Spraying of these VOCs on cucumber plants has been found to reduce whitefly infestation under field conditions [13]. Similarly, intercropping tomato plants with aromatic plants such as dill and coriander reduce whitefly visits in tomato plants. Whiteflies are repelled by several VOCs emitted by dill and coriander, thus highlighting the potential of VOCs emitted by companion plants in controlling whiteflies [14]. Furthermore, intercropping pepper plants with horticultural plants such as basil and French marigold protected pepper plants from aphids (*Myzus persicae*). Eugenol and (*E*)-β-farnesene emitted by these companion plants exhibited considerable repellent effects on *M. persicae* [15]. Moreover, these plant VOCs can be synthesized synthetically in laboratories and are also commonly used in agriculture. For instance, the application of (*Z*)-3-hexenyl acetate in hopyards improved the attraction of predatory mirids and anthocorids, while the application of methyl salicylate stimulated the attraction of geocorids and hoverflies [16]. Similarly, the application of synthetic herbivore-induced VOCs in commercial greenhouses using dispensers attracts the larval parasitoid *Cotesia vestalis*, causing a notable decrease in the occurrence of the Dimond back moth, *Plutella xylostella* [17]. Similarly, methyl salicylate and eugenol possess the ability to attract green lacewings and other predatory arthropods under field conditions [18, 19]. The application of elicitor compounds, such as jasmonates, to crops, including tomato, rice and soybean, has also been found to induce the production of VOCs attractive to parasitoids [20–22]. However, it is important to note that in many cases, similar experimental designs failed to yield positive results under field conditions [23]. In summary, evaluating the impact of companion plants on agricultural pests in intercropping systems and identifying potential behaviorally active VOCs emitted by companion plants offers an effective and sustainable approach to develop pest management strategies in agriculture.

The potential of VOCs for crop protection against biotic stressors has been demonstrated repeatedly under controlled environmental and field settings [10, 24, 25]. However, the application of VOCs under field conditions often results in untargeted effects, thereby limiting the effectiveness of VOC-based pest management strategies. For instance, some synthetic VOCs used as “alarm calls” for natural enemies may also be perceived by herbivores and increase their egg loading on crops [26]. Thus, a particular VOC can be strongly repellent to one herbivore species but highly attractive to another; hence, its usage may increase crop damage by secondary pests [27–29]. Boosting the emission of (*E*)-β-caryophyllene and α-humulene in maize plants by genetic transformation increased the attraction of entomopathogenic nematodes and reduced root damage by root herbivores, pointing to the potential agricultural application of these VOCs. However, it was reported that leaf herbivory concomitantly increased while seed germination, plant growth, and yield were compromised, therefore indicating the need for careful design to implement these approaches, such as targeting the release of VOCs in a tissue-specific manner [30–32].

To date, > 700 different plant VOCs have been identified, among which hundreds can be produced and emitted by a single plant species [33–35]. Although our understanding of their biological function has significantly increased in recent decades, especially under controlled environments and laboratory settings, there remains a need to understand their effects in natural settings and their potential ecological pleiotropic effects. This review provides comprehensive knowledge and analysis of the literature on the ecological pleiotropy of plant VOCs as well as its implications for their application in the sustainable management of crop health.

## What is ecological pleiotropy?

The term “Pleiotropy” was first proposed by the German geneticist Ludwig Plate in 1910 [36]. It is defined as the phenomenon in which a single locus controls two or more seemingly unrelated phenotypic traits [37]. The phenomenon of pleiotropy has garnered significant attention in the scientific community, particularly in relation to traits pertaining to growth, morphology, development, or fitness in animals [38]. A gene that regulates fly wing size also affects lifespan, or a gene responsible for the production of melanin is also involved in immune regulation [39–41]. Most of these pleiotropic effects were studied and characterized in controlled environments, where the organisms studied were isolated from other interacting species [42]. Later, by studying the effects and ecological functions of a single gene or trait, ecologists and evolutionary biologists coined the term of “Ecological Pleiotropy” to refer to a phenomenon in which a gene, a set of genes, or a trait influence multiple traits that affect an organism's fitness in different ecological contexts or to refer to those gene(s) or traits that affect several ecological processes [43]. Traits exhibiting ecological pleiotropy include body mass, phenology, color patterns and those affecting the rate of encounters with interacting species in the ecosystem [44]. For instance, it has been observed that genes play a regulatory role in controlling the aggression and migration behavior of birds, as well as influencing their immune response to disease [45]. In fish, a gene that regulates mate choice also affects disease resistance [46]. In insects, certain gustatory receptors regulate the behavioral responses of males and females to a putative inhibitory pheromone, as well as the production of this pheromone in males [47]. The emission of (*E*)-β-caryophyllene from Arabidopsis leaves has the capacity to repel *Diaphorina citri*; however, it serves as a defense mechanism against bacterial pathogens when released from flowers [48, 49]. In plants, genes that affect plant architecture and morphology are also ecologically pleiotropic. For example, the Teosinte glume architecture (TGA1) gene in maize affects ear number, tassel branch length, and ear length [50, 51]. The LAX PANICLE1 gene (LAX1) in rice has been found to have effects on plant branching, grain shape, and tiller number [52]. At the ecological level, pleiotropy also plays a significant role in the adaptation of plants to their environment and their interactions with other organisms in nature, as seen in examples such as the PROCERA gene in tomato [53], DELAYED FRUIT RIPENING (DFR) gene [54, 55] and GmTIFY10A gene in soybeans [56], which exhibited pleiotropic effects on multiple traits such as drought tolerance, fruit ripening, resistance to fungal infections and regulation of salt and drought stress.

## Evaluating ecological pleiotropy induced by the effects of VOCs

Several organisms can produce, perceive, and respond to VOCs [57]. Therefore, the impact of VOCs can lead to ecological pleiotropy, which can be classified into concurring pleiotropy and opposing pleiotropy, depending on the direction and magnitude of the effect [58, 59]. Concurring pleiotropy refers to a phenomenon in which a gene or a set of genes influence the multiple traits that interact positively with each other, resulting in an overall enhancement or reduction in the fitness of an organism. On the other hand, opposing pleiotropy occurs when a particular gene, group of genes, or trait has contrasting effects on fitness in different ecological settings, leading to ecological trade-offs. In the context of VOC-mediated ecological pleiotropy, the effects of a VOC on different traits combine synergistically to produce a greater fitness advantage than the sum of their individual effects (concurring pleiotropy) or can have contrasting effects on fitness in different ecological settings (opposing pleiotropy) (Fig. 2). Different examples of pleiotropy mediated by VOCs are listed in Table 1.

**Table 1:** Concurring and opposing pleiotropy of VOCs. The following abbreviations for the ‘Receivers’ of VOCs are used: Plant (P), herbivores (H), natural enemies (N), pollinators (O), microbes (M) and abiotic environment (A). The following abbreviations for the effect of the VOC are used: ( +) positive and (-) negative

Plant species	VOC	Type of pleiotropy	Interacting organisms	References
***Arabidopsis thaliana***	(*E*)-β-caryophyllene	+ H + M	*Diaphorina citri,* *Pseudomonas syringae*	[48, 49]
	(*E*)-β-farnesene	+ H –N	*Myzus persicae,* *Diaeretiella rapae*	[60]
***Biscutella laevigata***	β-ocimene	+ N –O	*Thomisus onustus,* *Apis mellifera,* *Halictus sp.*	[61]
***Chrysanthemum morifolium***	(*E*)-β-caryophyllene	+ N + N	*Arma chinensis*, *Trichogramma pretiosum*	[62]
***Capsicum annuum***	(*Z*)-3-hexenyl propanoate	+ H + N	*Aphelinus abdominalis, Aulacorthum solani*	[63]
***Chrysanthemum nankingense***	(*E*)-β-caryophyllene	+ A –H	*Spodoptera litura*	[64]
***Fagopyrum esculentum***	(*Z*)-3-hexenol	+ O + O	Bumblebees, honeybees, flies	[65]
***Gossypium hirsutum***	(*E*)-β-caryophyllene (GhTPS1)	+ H + N	*Apolygus lucorum,* *Aphis gossypii,* *Helicoverpa armigera,* *Peristenus spretus,* *Aphidius gifuensis,*	[66]
***Nicotiana attenuata***	Benzyl acetone	+ O + H	*Manduca sexta*, *Diabrotica undecimpunctata*	[67]
***Origanum vulgare***	Linalool	+ N + N	*Heterospilus prosopidis,* *Cotesia glomerata*	[68]
***Phaseolus vulgaris***	1-undecanol, (Z)-3-hexen-1-ol	+ M + H	*Pseudomonas syringae,* *Tetranychus urticae*	[69]
***Solanum lycopersicum***	Octyl acetate	+ H –N	*Encarsia formosa,* *Macrolophus pygmaeus*	[70]
	2-carene	–N + A	*Microplitis Croceipes*	[71]
	(*E*)-α-bergamotene	+ H + H	*Macrosiphum euphorbiae, Myzus persicae, Aphid Diaeretiella*	[72, 73]
***Solanum tuberosum***	β-caryophyllene	+ H + N + A	*Phthorimaea operculella*, *Trichogramma chilonis*	[74]
***Vitis vinifera***	Phenylacetonitrile	–H –H	*Sparganothis pilleriana, Lobesia botrana*	[75]
***Vigna radiata***	2-hexenal, Benzaldehyde	–H –H	*Callosobruchus chinensis,* *Oedaleus asiaticus,* *Angaracris barabensis* *Spilosoma obliqua*	[76–78]
***Zea mays***	(*E*)-β-caryophyllene	+ H + N	*Diabrotica virgifera, Spodoptera litura, Heterorhabditis megidis, Cotesia marginiventris*	[30–32]
	(*E*)-α-bergamotene	+ N –H	*Cotesia marginiventris,* *Spodoptera frugiperda*	[79, 80]
	(*E*)-β-caryophyllene, Ethylene	+ H –H	*Spodoptera littoralis,* *Diabrotica virgifera*	[28]

## Concurring pleiotropy

VOC-dependent concurring pleiotropy can be classified into two types, positive or negative pleiotropy, depending on the effects to the emitter and to the receiver. Positive concurring pleiotropy indicates that one organism trait positively affects multiple traits/phenotypes or ecological interactions. For example, VOCs emitted by plants can act both as attractants for natural enemies of herbivores and as repellents for the herbivores themselves. Thus, it can be observed that ecological pleiotropy presents a positive correlation when a particular trait produces a consistent effect on two or more functions within the ecosystem. There are several examples of positive concurring ecological pleiotropy for plant VOCs (Table 1). For example, limonene, which is emitted by citrus plants, has different functions, including repelling herbivores (+ H), attracting their natural enemies (+ N), increasing pollinator (+ P) visits, and protecting plants from pathogens (+ M) [81–83]. Moreover, (*E*)-β-caryophyllene, which is released by *Chrysanthemum morifolium* flowers, demonstrates attractive properties for multiple natural enemies (+ N, + N) [62] while repelling herbivorous moths and attracting their egg parasitoids in high-temperature stressed potato plants (+ H, + N) [74]. Similarly, (*Z*)-3-hexenol, which is released by the buckwheat plant *Fagopyrum esculentum*, may attract several species of pollinator bees (+ O, + O) [65]. In tomato plants, the release of (*E*)-α-bergamotene has been shown to have a repellent effect on several herbivorous insects (+ H, + H) [73]. Last, the release of benzyl acetone from tobacco plants has the dual function of attracting pollinators and repelling herbivores (+ O, + H) [67].

Negative concurring pleiotropy results when a single gene or trait negatively impacts multiple traits/phenotypes or ecological interactions. For example, in maize plants, the emission of linalool has been found to attract multiple herbivore species, such as stink bug (*Dichelops furcatus*) [84], western corn rootworm (*Diabrotica virgifera*), northern com rootworm, (*Diabrotica barberi*) [85] and fall armyworm, (*Spodoptera frugiperda*) [86], thus adversely affecting plant growth and reproduction. Similarly, in citrus plants, methyl salicylate emitted from *Diaphorina citri*-infested plants reduced the performance and attraction of the predator ladybird beetle *Propylaea japonica* and promoted *D. citri* infestations [87]. The emission of (*Z*)-3-hexen-1-ol by the medicinal plant *Glycyrrhiza uralensis* attracts the leaf chewing beetle *Diorhabda tarsalis* [88], but this compound possesses repelling properties to parasitic wasp *Cotesia glomerata* cabbage plants infested with *Pieris brassicae* [89]. Furthermore, in Arabidopsis, the overexpression of ZmLOX6, which produces pentyl leaf VOCs, affects plant hormonal networks and plant growth while increasing the attraction of aphids [90]. In summary, exploring the negative pleiotropy of plant VOCs in the context of interacting organisms might provide valuable insights to develop strategies for sustainable agriculture, pest management, and conservation. The identification of genetic variants that influence plant–insect interactions can offer the development of novel plant breeding tactics to enhance resistance to agricultural pests while minimizing negative side effects on other aspects of plant biology.

## Opposing pleiotropy

**Fig. 1:**
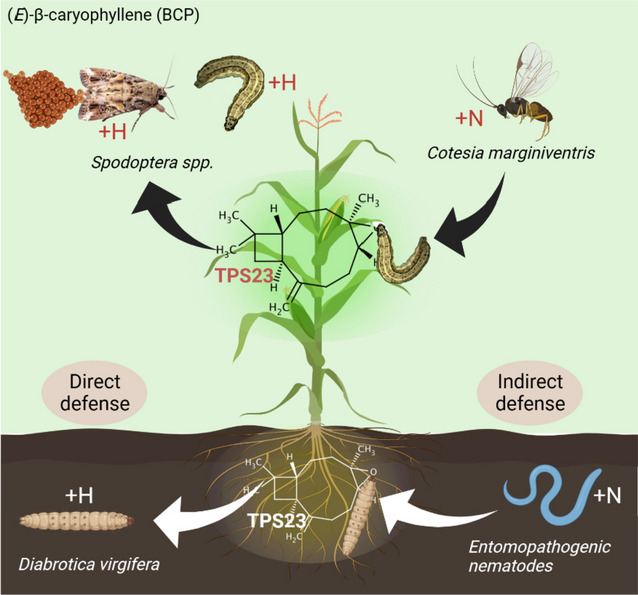
Pleiotropic functions of (*E*)-β-caryophyllene in maize plants and several interacting organisms, including leaf herbivore (*Spodoptera littoralis*) larval parasitoid (*Cotesia marginiventris*), western corn rootworm (*Diabrotica virgifera*) and entomopathogenic nematode (*Heterorhabditis megidis*). The figure was created with BioRender (biorender.com/)

Opposing pleiotropy occurs when a single VOC has multiple effects (negative (-) and positive ( +) on plant fitness as well as on the interacting organisms. A plant VOC might attract some beneficial organisms, such as pollinators or natural enemies, but can also attract harmful organisms, such as herbivores (Table 1). Methyl jasmonate has the potential to attract natural herbivore enemies, including the egg parasitoid *Trichogramma pretiosum* (+ N), while inducing plant defenses that can impair plant growth (+ H, P) [91]. Similarly, octyl acetate emitted from tomato plants repels both herbivores and natural enemies (+ H,–N) [70]. Similarly, another VOC, (*E*)-α-bergamotene, released from maize plants has the capacity to attract both herbivores and natural enemies (–H, + N) [79, 80] (Fig. 1). Moreover, β-ocimene released from mustard flowers has shown a tendency to repel honeybee pollinators but at the same time attracts herbivorous natural enemies such as crab spider (*Thomisus onustus*) (–O, + N) [61].

## Determinants of VOC-mediated ecological pleiotropy

VOC-mediated ecological pleiotropy can be affected by different factors in a similar manner, as the ecological function of VOCs is modulated by several factors [92, 93]. The main factors include i) the chemical properties and structure of a VOC, which can influence its ability to interact with different organisms and perform different functions [94]; ii) environmental abiotic factors such as light, temperature and humidity as well as biotic factors such as herbivores and microorganisms, which can all influence the emission and ecological functions of plant VOCs [74, 95–97]; iii) coevolutionary interactions between plants and their associated organisms, which can influence the selective pressures on VOC production and their ecological functions [98]; iv) the concentration of a VOC can affect its perception and response by different organisms [99–101]; v) the physiological status of the plant, such as nutrient uptake and stress level, can also affect the VOCs production and their ecological effects [102, 103]; vi) the genetic variation within a plant population can affect the types and amounts of VOCs produced, which can influence the ecological pleiotropy of these VOCs [104]; and vii) the developmental stage of a plant can affect the types and amounts of VOCs it produces, as well as their ecological effects [105].

In general, the biodiversity levels in a habitat, including genetic diversity, species diversity and ecosystem diversity, can all influence the formation of ecological pleiotropy for a plant VOC trait. Specifically, the emitter and receiver of a plant VOC trait, as well as the timing and location of its production, can have significant implications for its ecological pleiotropy, ultimately affecting plant fitness.

### The diversity of plant VOC receivers determines the magnitude of ecological pleiotropy

The organisms that can use or are impacted by plant VOCs are called the “receivers” [101]. The species diversity of the “receivers” in an ecosystem not only affects the structure of the food web but also determines the number of nodes in the infochemical web connected by certain plant VOCs. Upon reception, VOCs can serve either as precursors for the biosynthesis of other compounds [106] or can function as signaling molecules that regulate the physiology of the receiver organisms [107]. These two processes can be modulated depending on the genetic diversity of the interacting organisms, especially of the receiver organisms [108].

Moreover, it is well established that plants themselves can also be “receivers” by actively absorbing other VOCs from the environment or other emitting plants. These VOCs are metabolized upon uptake in the plant cell. Methanol, for instance, can be oxidized to formaldehyde [109]. Dimethyl disulfide can be incorporated into plant proteins, which can promote plant growth [110]. *Ent*-kaurene acts as a precursor for gibberellin biosynthesis [111]. (*Z*)-3-hexen-1-ol can be glycosylated into (*Z*)-3-hexen-1-yl vicianoside in tomato plants, which boosts plant resistance against herbivores [112]. The (*Z*)-3-hexenyl acetate is metabolized into (*Z*)-3-hexenol by Arabidopsis plants, which is facilitated by the carboxylesterases AtCXE5 and AtCXE12, contributing to the esterase activity of (*Z*)-3-hexenyl acetate in leaves. However, the mechanisms by which plants metabolize these VOCs are still unknown [113]. Exposing Arabidopsis plants to (*E*)-2-hexenal suppresses root growth, potentially via affecting the redox status of root cells; thus, this highly reactive electrophile VOC may be involved in a signaling event in plant cells [114].

In addition to plants, other organisms have the capacity to act as VOC receivers. For instance, insects use their chemosensory system to perceive plant VOCs during forage and plant host selection. Certain insects have evolved to detect specific VOCs emitted by plants as cues to locate their hosts or prey. However, these same VOCs can also attract predators or parasitoids of the insect, resulting in a trade-off between the benefits of finding food or shelter and the risks of being attacked [115]. For instance, (*Z*)-3-hexenol) released by tomato plants has numerous effects, including avoiding the whitefly [116] and attracting the parasitic wasp *Encarsia formosa* [70]. It is interesting to note that the same VOC released from different parts of plants can regulate various ecological targets, as seen with farnesene in rape leaves attracting aphid flies and promoting parasitic wasp olfactory response, whereas the same VOC can also be continuously released on rape flowers to regulate the pollination behavior of bumblebees [117, 118]. These factors may increase the complexity of VOCs pleiotropy in regulating the behavior of insects when interacting with their host plant.

In a comparable manner, diverse herbivorous species may exhibit distinct reactions to the same VOCs released by plants, hence influencing their feeding patterns and interactions with other organisms within the ecosystem. A number of herbivores may be attracted to certain VOCs as a food cue, while others may be deterred by the same compounds as a defense mechanism. For example, (*E*)-β-caryophyllene provides constitutive foliar defense against *Spodoptera species,* and this terpene is also released by roots after specialist *Diabrotica virgifera* damage. Additionally, it has been suggested that (*E*)-β-caryophyllene can attract natural enemies of both herbivores: entomopathogenic nematodes below ground and parasitic wasps above ground [30–32]. Previously, it was reported that *D. virgifera* 2^nd^ instar larvae showed attraction toward maize roots due to the emission of (*E*)-β-caryophyllene, but their neonates did not exploit (*E*)-β-caryophyllene in locating maize roots [119]. Furthermore, *D. virgifera* larvae showed a stronger growth rate on roots that were damaged by conspecific larvae, but their performance was decreased on roots of plants whose leaves had been attacked by larvae of the moth *Spodoptera littoralis*. They also identified that (*E*)-β-caryophyllene was induced by *D. virgifera* larvae and ethylene, which was suppressed by *S. littoralis* feeding on leaves, showing the two signals exploited by *D. virgifera* larvae to locate plants [28]. Indole, which is an essential herbivore-induced VOC, primes maize plant VOCs signaling [120] and regulates different aspects of microbes physiology, such as bacterial spore formation, drug resistance, biofilm formation and maintenance, plasmid stability and virulence factor production in indole-synthesizing bacteria [121]. Thus, affecting insect-parasitoid interactions, such as exposing *Spodoptera littoralis* caterpillars to indole, results in a reduction in their attractiveness to the endo-parasitoid *Microplitis rufiventris*, indicating that VOCs in-take by herbivore is not the endpoint for their ecological functions [115].

In addition to the chemosensory systems, other physiological systems or phenotypes of insects can also be directly impacted by plant VOCs. During herbivory, VOCs in plant tissues can be ingested by the insect guts, thereby exerting a direct influence on the physiology and performance of those herbivores [122]. For instance, dimethyl disulfide and dimethyl trisulfide have been reported to exert insecticidal neurotoxicity to insects through mitochondrial dysfunction [123]. Similarly, the impact of (*E*)-β-farnesene on the development of *Spodoptera* larvae has also been reported [124]. Exposing *Helicoverpa armigera* to limonene, nerolidol, 2-heptanone and cis-3-hexenyl acetate dramatically increased larval tolerance to the carbamate insecticide methomyl, which is likely caused by the upregulation of the transcription and enhanced activity of metabolite enzymes, including P450 monooxygenase, glutathione-*S*-transferases, and carboxylesterases [125]. A similar phenomenon is also observed in *Spodoptera litura* exposed to VOCs from tomato [126].

Microorganisms can also be recipients of plant VOCs, which can inhibit the growth and reproduction of phytopathogenic bacteria, fungi, and viruses. For instance, the synthesis of geraniol by OsTPS21 in rice increased resistance against *Xanthomonas oryzae,* which is responsible for rice bacterial blight. Geraniol negatively affected the growth of *X. oryzae* by suppressing cell division-related genes in *X. oryzae* [127]. On the other hand, phyllospheric and rhizosphere microorganisms, many of which are beneficial to plants, can all be affected by plant VOCs [128]. For example, the roots of *Carex arenaria* plants infected with a fungal pathogen (*Fusarium culmorum*) altered the VOCs profile and bacterial interactions. These fungal-induced VOCs of *C. arenaria* were found to stimulate the attraction of beneficial bacteria with antifungal properties from longer distances (12 cm), indicating that plants have the ability to alter VOCs compositions in order to attract microorganisms following variable soil environments [129].

### The ecological function of a single VOC is largely modulated by the blend of co-emitted volatiles

Plants often release a blend of different VOCs that can quantitatively and qualitatively vary depending on the ecological context [130, 131]. It is commonly found that multiple VOCs can affect a receiver organism in a similar manner [132]. In tobacco plants, the floral release of β-pinene, methyl salicylate, linalool and limonene can all increase the attraction of predators such as *Coccinella septempunctata*, *Harmonia axyridis,* and *Hippodamia variegate* and parasitoids such as *Aphidius gifuensis* [133]. In cotton plants, 3,7-dimethyl-1,3,6-octatriene and (*Z*)-3-hexenyl acetate are induced by *Helicoverpa armigera*, and these two VOCs can both elicit an electroantennogram response in the larval endo-parasitoid *Microplitis mediator* and attract this naturally in the olfactometer assay [134]. On the other hand, certain VOCs have the ability to interact and modulate the physiological responses of the receiver organism. The capability of simultaneously recognizing multiple VOCs is a common characteristic of the chemosensory system in insects. Insects have chemosensory receptors in their antennae, by which they perceive different VOCs and trigger mosaic signaling, which is further integrated into the respective chemosensory circuit [135]. Meanwhile, the recognition of VOCs by chemosensory receptors is not strictly 'yes or no', but often in the way of one receptor binding a wider range of compounds, although the response intensity to those VOCs can be different [136]. Thus, the chemical composition of the VOC blends can significantly impact the searching and selection of insects for host plants [137]. For instance, it was observed in olfactometer tests that the female ladybird predator *Stethorus punctum picipes* showed attraction toward the VOCs mixture (cis-3-hexen-1-ol, cis-3-hexenyl acetate and methyl salicylate) but not to the individual VOC. Additionally, it was found that male predators did not display any attraction to the VOCs mixture. However, in open field tests using sticky cards, the predators demonstrated their attractiveness to the VOCs mixture and to MeSA alone but not to cis-3-hexen-1-ol and cis-3-hexenyl acetate [138]. Overall, the ecological pleiotropy of a plant VOC is affected by the specific volatile background in which it is presented.

### Biosynthesis of plant VOCs is the driving force shaping the functional pleiotropy of VOCs in nature

The process of plant VOCs biosynthesis can affect the diversity and specificity of VOC production. Different biosynthetic pathways may lead to the production of similar or different VOCs with distinct ecological roles [139–141]. Generally, plant VOCs are biosynthesized in four major pathways, namely, the shikimate/phenylalanine, mevalonic acid (MVA), methylerythritol phosphate (MEP) and lipoxygenase (LOX) pathways [8]. Several of the enzymes responsible for VOCs synthesis are multifunctional. For instance, TPS33 proteins convert (*Z,Z*)-farnesyl diphosphate into multiple terpene products, such as β-curcumene, β-acoradiene, *cis*-α-bergamotene, and α-cedrene [142]. In maize, TPS10 proteins convert farnesyl diphosphate into different terpenes, such as (*E*)-α-bergamotene, sesquisabinene, (*E*)-β-farnesene, zingiberene, β-bisabolene, and sesquiphellandrene [79]. Apparently, since different VOCs produced by the same enzyme may have different ecological functions on a variety of receivers, ecological pleiotropy for the genes producing these enzymes could be common for plants [143]. This phenomenon is important, especially in the genetic manipulation of genes responsible for plant VOC biosynthesis.

Furthermore, following the biosynthesis and emission of VOCs, structural alterations may occur in response to environmental factors. For instance, ozone may degrade plant-released VOCs, which can further influence plant–insect interactions [144–148]. The emission of several VOCs, such as α-pinene, p-cymene, phenol, benzaldehyde and anisaldehyde, by *Brassica nigra* flowers is adversely affected by ozone, which in turn strongly impairs the *Bombus terrestris* host location of Bombus terrestris [145]. Similarly, exposing *Brassica oleracea* plants to moderate atmospheric ozone levels leads to the complete degradation of common constitutive (α-pinene, sabinene, β-myrcene and limonene) and herbivore-induced VOCs (α-thujene, α-pinene, β-myrcene and limonene); however, the orientation behavior of natural enemies (*Cotesia plutellae*) toward damaged plants remains unaffected [148]. These findings indicate that the elevated ozone levels in the surrounding environment can have a significant impact on plant–insect interactions by influencing both VOCs and VOC-elicited responses.

UV light can also influence VOCs, as depicted by a previous study showing that espousing *Prunus persica* fruits and leaves with UV-B irradiation decreased the emission of the monoterpene linalool by 60% [149]. Linalool regulates plant–insect interactions in multiple manners [94, 150, 151]. The degradation of this VOC under UV light can influence the orientation of these insects toward their host plant.

### Ecological functions of VOCs are determined by spatial–temporal factors

The timing and location of VOC emission can have an impact on the interactions with different organisms and the specific functions they perform [59]. The most exemplary instances within this particular context have been thoroughly examined, with due consideration given to the various stages of plant growth and reproduction. Plant flowers emit distinct VOCs from leaves, which attract pollinators [152]. Some of these volatiles may in turn repel herbivore caterpillars [153], attract various beneficial community members, including carnivorous arthropods and parasitic nematodes [154, 155], and might be perceived by neighboring plants [156]. For instance, β-elemene, which is only emitted by *Elsholtzia rugulosa* flowers, attracts honeybees [152], whereas (*Z*)-9-octadecenal and octadecanal, which are specifically released by *Solanum carolinense* flowers, influence the attraction of bumblebees, honeybees, and solitary bees [157]. The emissions of VOCs from flowers and from undamaged and herbivore-damaged leaves often show distinct diurnal or nocturnal patterns [158]. This may be the result of circadian regulation of substrate availability, transcription or enzyme activity [159]. Zhou et al. demonstrated the pleiotropic role of (*E*)-α-bergamotene that was potentially regulated by the circadian system in wild tobacco. Its release by flowers at night enhances the success of adult *Manduca sexta* moth-mediated pollination during the night flowering of wild tobacco. Moreover, its discharge in herbivory-damaged leaves in the daytime may act as an indirect defense mechanism against *M. sexta* larvae [158]. Similarly, another study in wild tobacco found that the green leaf volatile (GLV) biosynthetic enzyme HYDROPEROXIDE LYASE is transcriptionally regulated by the circadian clock; accordingly, the GLV aldehyde pools in leaves peaked at night [160]. The available information indicates that spatial–temporal emission of VOCs may be coordinated to the circadian rhythm for locomotion of insect receivers. However, the underlying mechanism responsible for regulating this phenomenon remains elusive. Furthermore, there is a need to explore the impact of spatial–temporal emission of VOCs on ecological pleiotropy in the natural environment as well as its influence on the fitness of plants in their habitats, which remains to be studied.

## The ecological pleiotropy of VOCs in sustainable management of crop health

**Fig. 2:**
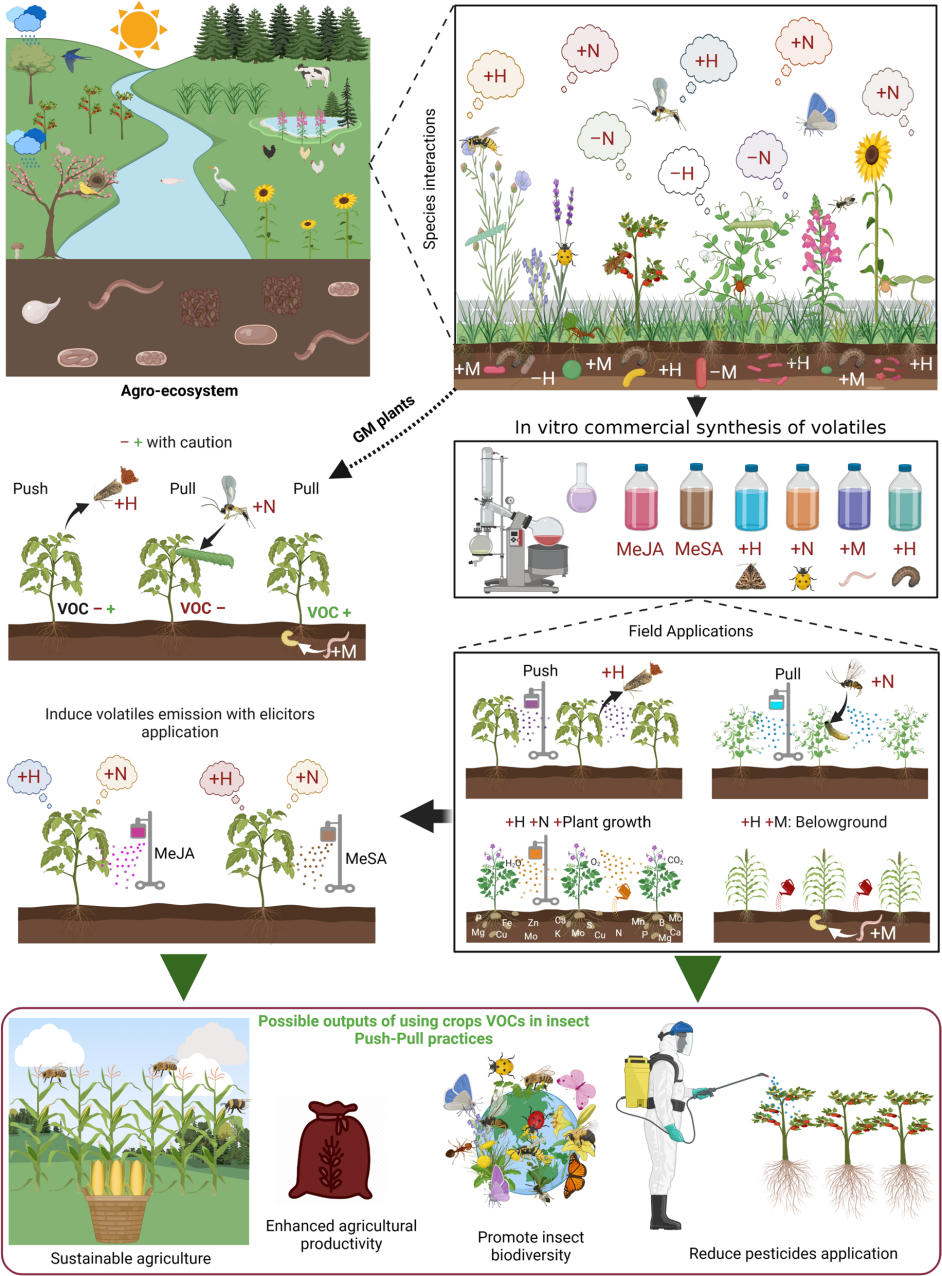
A general overview showing the pleiotropic functions of VOCs in agricultural pest management strategies. In the context of agro-ecosystems, the interactions between species carry significant implications for the overall health, productivity, and sustainability of the system. Insect species engage in a variety of relationships, including mutualism, competition, predation, herbivory, parasitism, and commensalism, with these interactions primarily regulated by the emission of plant VOCs. The application of synthetic VOCs in vitro can serve to either repel or attract targeted insect species. Additionally, the development of transgenic plants exhibiting suppressed or enhanced emission of specific VOCs or the induction of these VOCs through the use of plant defense elicitors such as MeSA or MeJA presents a promising avenue. Implementing VOCs-based pest management strategies in agricultural landscapes holds the potential to foster sustainable agriculture. This approach not only enhances crop productivity but also promotes beneficial insect biodiversity, ultimately mitigating the adverse health effects associated with pesticide applications. The figure was created with BioRender (biorender.com/)

To date, VOCs are employed in the agricultural sector in three distinct forms. One way of using VOCs in agriculture involves the manipulation of VOCs in crops. This includes the genetic modification of crops to enhance or suppress the production of specific VOCs [26, 161–163] (Fig. 2). These plants have been used in agriculture as traps or as repellent plants in push–pull systems [164]. Although there are some successful cases of VOC-based pest management [165], compared to other agrochemicals such as botanicals, the exploration for the potential of VOCs in agricultural applications remains very limited [10, 24, 166, 167]. The second way to utilize VOCs in agriculture is by regulating VOC blends in agroecosystems (Fig. 2). This entails the use of techniques such as intercropping [168–171] or crop rotation [172–174] to alter the mix of VOCs released by plant communities in a given area. By strategically planting crops that emit different VOCs, the behavior of certain pests and/or certain natural enemies can be modulated (Fig. 2). A third form involves the deployment of plant VOCs in their pure forms (produced in laboratories) (Fig. 2). In this context, VOCs can act as repellents for herbivores to reduce insect populations [18] or as attractants for herbivores to monitor pest populations [175]. Moreover, these compounds act as plant defense elicitors to induce pest resistance in crops [24], as botanical pesticides to suppress herbivores and pathogens [176], or as insect attractants for recruiting natural enemies [12].

### Importance of the identification of VOC-associated pleiotropic effects

Compared to other crop traits, such as product quality and crop productivity, VOC-associated traits were very likely neglected during crop domestication and are not currently the main target of crop breeding [177]. Thus, understanding how plant domestication impacts plant VOCs deserves further research efforts [178]. For instance, it has been reported that wild potato accessions have shown attractiveness to *Diaeretiella rapae* parasitoids and stronger repellency to adult aphid herbivores. Additionally, chemical investigations have revealed significant variations in VOCs between wild and cultivated potato accessions [72]. In tomato plants, natural enemies such as *Jalysus wickhami* predators and *Cotesia congregata* parasitoids prefer HIPVs of wild tomatoes over HIPVs of cultivated tomato plants [179]. In our recent study (Munawar et al.), we found that the addition of herbivory on wild potato leaves can pull predators back from flowers to leaves, which is mediated by the emission of plant VOCs. The same phenomenon was also observed in cultivated potato species [180]. It is likely that many VOC traits having pleiotropic ecological functions for crop fitness have been lost during domestication or breeding. Clarifying the effect of domestication or breeding on crop VOC-mediated insect-plant interactions will be helpful to ‘resurrect’ these functional VOC-associated traits.

A more precise genetic modification of the emitters for VOCs, either through conventional breeding or via modern gene editing tools, can be used to avoid the 'off-target effects of VOCs' observed in many previous studies [9, 181]. First, the genetic manipulation of multifunctional enzymes catalyzing the biosynthesis of VOCs may be restricted to loci that influence the production of a specific VOC rather than all products [142]. Second, to avoid the excessive consumption of photosynthetic resources and autotoxicity effects resulting from overproduction and overaccumulation of VOCs, the overexpression of their metabolic enzyme-encoding genes should be carefully engineered [182]. Moreover, the identification of regulatory elements, including transcription factors and cotransforming them with enzymes, can help in achieving well-controlled spatiotemporal emission of VOCs [140]. Third, altering the response of receivers to VOCs by editing the receptors in crops [183] may help to avoid the negative effect of these chemicals on their growth, reproduction, and quality. Moreover, as Wright (1968) concluded, 'Pleiotropy has a broader meaning in population genetics than in physiological genetics' [184], increasing the intraspecies diversity in VOC production for crops may also serve as a viable approach for improved pest management, specifically if this level of diversity does not influence crop growth, productivity, and product quality. In other words, the next generation of “Push–Pull” can also be fulfilled at the intraspecies level of crops where different individuals of crops emit a mosaic of functional VOCs.

### Unrevealing the interspecies infochemical network in crops promotes the usage of VOCs in agro-ecosystems

Clarifying the ecological pleiotropy of crop VOCs in the open field has posed a significant challenge for the ecologist, not only because the VOCs are in a changeable state but also due to the prevalence and frequency of species migration. With the innovation for large-scale real-time detection and analysis of VOCs in the field [185–187], observing the structure of this infochemical network as well as its dynamic changes has become possible. Functional VOCs play a crucial role in agro-ecosystems by establishing connections between various species referred to as "nodes" and offering valuable insights into their interactions [188]. By conducting a comprehensive search for the “hub” receiver species and clarifying the key VOCs sent or received by them, it becomes possible to unravel that the changes in a single VOC may “rewire” one or multiple ecological networks among species, which holds significant implications for the application of VOCs in modern agriculture. Overall, in pest management, it is reasonable to focus on the “hub” species in the infochemical network having a significant effect on crop health, which include pests related to the economic threshold of crop production or ecological risk [189]; key beneficial organisms such as parasitoids, predators, and entomopathogenic microorganisms; dominant pollinators; and other crop species or companion plants. Those infochemical branches with minor effects on crop health and the stability of agro-ecosystems can be neglected.

When introducing an external VOC into a habitat, its potential impacts on the local infochemical network also need to be considered (Fig. 2). It is crucial to assess the adverse impact of utilizing VOCs as attractants or repellents for herbivore targeting, particularly in relation to their effects on natural enemies, pollinators, and other beneficial organisms. Moreover, it is also important to evaluate the attractiveness of these VOCs for other herbivore species to avoid their ecosystem disfunctions [24, 190, 191]. For instance, the hyperparasitoid *Lysibia nana* preferred VOCs emitted by *Brassica oleracea* in response to *Pieris brassicae* caterpillar parasitized by the host *Cotesia glomerata* over the VOCs emitted by *Brassica oleracea* in response to *Pieris brassicae* caterpillar parasitized by the non-host *Hyposoter ebeninus* [192]. Furthermore, these VOCs have the ability to attract different insect species, leading to interspecies carnivalism. Thus, elucidating the changes in species interactions within the habitat resulting from the application of external VOCs may avoid the strengthening of unexpected ecological interactions negatively affecting the fitness of crops [24].

## Data Availability

Data sharing is not applicable to this article, as no datasets were generated or analyzed during the current study. Instead, it comprehensively surveys and analyzes literature, studies and published works in the field. All information, data and references cited in this review paper are duly acknowledged and properly cited within the text and in the reference list.
